# Morning cortisol and central adrenal insufficiency: new thresholds from low-dose ACTH test and second-generation assay

**DOI:** 10.1007/s40618-026-02807-5

**Published:** 2026-01-31

**Authors:** Valentina Gasco, Fabio Bioletto, Davide Lucisano, Davide Camoirano, Daniela Cuboni, Emanuele Varaldo, Michela Sibilla, Luigi Simone Aversa, Francesca Mocellini, Alessandro Maria Berton, Nunzia Prencipe, Ezio Ghigo, Mauro Maccario, Silvia Grottoli

**Affiliations:** Department of Medical Science, Division of Endocrinology, Diabetes and Metabolism, University of Turin, Turin, Italy

**Keywords:** Cortisol, Low-dose ACTH test, Secondary adrenal insufficiency, Sensitivity, Specificity, Diagnosis

## Abstract

**Purpose:**

According to current guidelines, a morning serum cortisol < 30 µg/L confirms central adrenal insufficiency (CAI), whereas a value > 150 µg/L rules it out. However, these thresholds are based on older assays, and intermediate values require further testing. Newer, specific monoclonal antibody immunoassays may have lower diagnostic thresholds. This study aimed to identify morning cortisol cut-offs with ≥ 95% specificity or sensitivity (SP/SE) to determine which patients may safely avoid ACTH stimulation testing, using a second-generation immunoassay.

**Methods:**

We retrospectively evaluated 435 adults (236 males; overall median age 58.5 [IQR 20.3] years) with pituitary disorders. Based on the 1 µg ACTH test, patients were classified as having or not having CAI using a peak cortisol cut-off of 180 µg/L (guideline) or 127 µg/L (Roche Elecsys^®^ Cortisol II–based studies).

**Results:**

With the 180 µg/L threshold, a morning cortisol ≤ 80.8 µg/L best predicted CAI (SE 37.1%, SP 95.2%), while > 144.0 µg/L best excluded it (SE 95.2%, SP 29.1%). Using the 127 µg/L threshold, a value ≤ 60.9 µg/L best predicted CAI (SE 54.7%, SP 96.3%), whereas > 141 µg/L (SE 96.2%, SP 21.2%) best ruled it out.

**Conclusions:**

We identified updated morning cortisol thresholds, specific to a second-generation immunoassay, that accurately predict ACTH test results and may streamline the diagnostic workup of suspected CAI. Based on these data, we propose a refined diagnostic algorithm.

## Introduction

Central adrenal insufficiency (CAI) results from impaired ACTH secretion due to hypothalamic or pituitary damage, leading to cortisol deficiency [1–4]. Its symptoms—fatigue, weight loss, hypotension—are often nonspecific and chronic, though acute adrenal crises may occur, presenting with severe hypotension, abdominal pain, and hyponatremia [1–4]. Due to its subtle presentation, CAI is frequently diagnosed late, and a high index of suspicion is essential [5, 6], as delayed treatment can be fatal [3–9]. However, misdiagnosis and unnecessary glucocorticoid therapy also carry serious risks [10, 11] making diagnostic accuracy crucial [6].

Once CAI is suspected, a variety of tests may be used to evaluate adrenal function [12]. A stepwise approach helps maintaining a costeffective screening and diagnosis.

Endocrine Society guideline for the diagnosis of hypopituitarism recommends measuring serum cortisol levels at 8–9 AM as the first-line test to diagnose CAI, suggesting that a serum cortisol level < 30 µg/L is indicative of CAI and a serum cortisol level > 150 µg/L likely excludes CAI. When morning serum cortisol values range between 30 and 150 µg/L, the Endocrine Society guideline recommend performing a stimulation test to diagnose CAI [12], using either an insulin tolerance test (ITT) or an ACTH stimulation test (in either its low-dose or standard-dose form) [12]. The ACTH stimulation test, which has been validated against the ITT—the ‘gold standard’—is a reliable tool for evaluating patients with suspected CAI, regardless of the underlying cause, as it is safer, more convenient, and free of contraindications [13]. Cortisol levels ≤ 180 µg/L after stimulation are indicative of CAI irrespective of the stimulation test employed [12].

Certainly, morning serum cortisol level represents a simple and cheap test for the evaluation of the HPA axis function, but there are no currently strong data about which morning serum cortisol concentration predicts a normal cortisol response to stimulation tests and, therefore, the morning baseline cortisol cut-offs that identify or exclude the diagnosis of CAI are supported by a very low-quality evidence, as clearly indicated by the guidelines themselves.

In the literature, several studies have aimed to identify morning baseline cortisol levels that can predict the response to the ACTH test. However, most of these studies are focused on the standard-dose test (250 µg) [14–20], while studies specifically oriented towards identifying such cut-off values for the low-dose ACTH test (1 µg) [21, 22] are considerably more limited. However, it is important to consider that the cortisol cut-off values, both basal and stimulated, recommended by guidelines and earlier studies, were derived from outdated cortisol assays. Moreover, the results largely depend on the sensitivity and specificity of the antibodies used in the selected assay. A major limitation is that these antibodies may cross-react and bind to antigens other than the intended target [23, 24]. In this regard, in recent years, studies have evaluated various second-generation cortisol immunoassays employing more specific antibodies and have proposed lower cortisol cut-off values for the ACTH test, ranging from 127 to 167 µg/L [25–31], which are below the previously recommended threshold [12]. Multiple cortisol cut-offs have been proposed in the literature for the Roche Elecsys^®^ Cortisol II assay, reflecting its lower cross-reactivity and reduced cortisol values compared with older methods. Among these, the 127 µg/L threshold—supported by Kline et al. [25] and Raverot et al. [26]—is the most robust and clinically meaningful. However, to our knowledge, no studies have evaluated the morning basal cortisol level that best predicts the response to the stimulation test using these new ACTH test response cut-offs.

The aim of this study was to identify the morning serum cortisol cut-off able to predict the response to a low-dose ACTH test in the diagnosis of CAI, in order to increase the quality of evidence on this topic. To this end, we used the traditional cortisol cut-off of 180 µg/L, still recommended by current guidelines, and also assessed basal cortisol performance in diagnosing CAI using 127 µg/L as pathological threshold for the 1 µg ACTH test, as proposed by recent studies employing the same assay method [25, 26, 28].

## Materials and methods

We retrospectively included all adult patients with history of pituitary disease who were referred to the Neuroendocrinology Clinic of our Center from 01.01.2015 to 31.12.2024 to be tested for HPA axis function.

The exclusion criteria included critical illness, nephrotic syndrome, severe liver disease, pregnancy, the use of enzyme-inducing drugs (such as oral estrogens or antiepileptic drugs), or any medications that could potentially interfere with the HPA axis, including chronic glucocorticoid therapy or immunotherapy.

We measured serum cortisol levels following an intravenous injection of 1 µg of ACTH (Synacthen ^®^) at time 0, with samples taken every 30 min from 0 to 60 min. When available, data on blood glucose and sodium levels measured concurrently with the ACTH test were also collected.

After an overnight fast, the test began in the morning at 8.00–8.30, 30 min after a peripheral venous catheter had been placed into a forearm vein that was kept patent by slow infusion of isotonic saline.

All subjects gave their informed consent to participate in the study, approved by the Local Ethics Committee (cod. 0040828).

Serum cortisol levels (µg/L; 1 µg/L = 2.759 nmol/L) were determined by a competitive electrochemiluminescence immunoassay (Roche Elecsys^®^ Cortisol II) automated on Cobas e601 instrument (Roche Diagnostics GmbH, Germany). Analytical sensitivity was 0.18 µg/L. Intra- and inter-assay precision ranged from 3.0% to 5.7% and from 2.4% to 6.2%, respectively.

All other biochemical variables were assayed in plasma or serum using routine automated methods in our Central Laboratory (Beckman Coulter AU automated chemistry analyzer), with internal quality controls in place according to laboratory standards.

All samples from a single subject were analyzed together. Continuous variables were expressed as median and interquartile range [IQR] due to non-normal distribution, while categorical data were expressed as percentages. Normality was assessed using the D’Agostino-Pearson test. Statistical analysis was performed using Friedman’s ANOVA and Kendall’s concordance test for comparing multiple dependent samples, and the Wilcoxon matched-pairs test for comparing two dependent samples. The Mann-Whitney U test was applied to compare independent samples. Correlations were assessed using Spearman’s rank correlation test. The Chi-squared test was used to determine if there was a significant difference between the observed and expected frequencies in one or more categories. Finally, a multivariate logistic regression model was built to predict CAI, using as covariates those features whose association with HPA impairment was demonstrated in univariate analysis or considered biologically plausible. A stepwise backward selection algorithm was applied to exclude variables that did not show an independent meaningful association (p value > 0.25) with the outcome of interest.

For the definition of CAI and for the subsequent analyses, we considered the ‘classical’ cut-off suggested by the guidelines—a peak cortisol response to the 1 µg ACTH stimulation test of ≤ 180 µg/L [12]—as well as a lower threshold of ≤ 127 µg/L, as proposed by more recent studies that employed the Roche Elecsys^®^ Cortisol II assay, the same method used in our study [25, 26, 28].

By using the Receiver-Operating Characteristic (ROC) Curve analysis, we probed the clinical utility of morning serum cortisol levels in the diagnosis of CAI. First of all, we identified the best cut-off for morning serum cortisol that predicts a pathological response to ACTH test. The best cut-off in a ROC curve is the closest point to that with the theoretical maximum of both sensitivity (SE) and specificity (SP). The area under the ROC curve (ROC AUC) represents the probability of correctly distinguishing between affected and non-affected individuals. Subsequently, we tried to identify the cortisol cut-off that identifies with a SP ≥ 95% those patients who should not undergo the stimulation test, because certainly affected by CAI or, conversely, that identifies with a SE ≥  95% those patients who should not be tested because certainly healthy.

For each identified cut-off we also estimate the positive predictive value (PPV), the negative predictive value (NPV), the positive likelihood ratio (LHR+), and the negative likelihood ratio (LHR-). Statistical analysis was performed using MedCalcTM^®^ (Statistical Software version 20.007, MedCalc Software Ltd, Ostend, Belgium).

## Results

The study population consisted of 435 patients (236 males; overall age 58.5 [20.3] years (yrs); overall BMI: 27.4 [6.7] kg/m^2^). Patients’ clinical characteristics are reported in Table 1.

**Table 1:** Characteristics of the entire cohort of patients with a history of hypothalamic-pituitary disease, as well as those with (HYPO-HPA) or without (NORMO-HPA) central adrenal insufficiency, defined by a cortisol peak ≤ 180 µg/L, or ≤ 127 µg/L during a low-dose ACTH stimulation test (statistically significant differences between the HYPO-HPA and NORMO-HPA groups are indicated in bold). Unless otherwise specified, data are presented as median and interquartile range (in squared brackets). (BMI: body mass index; IFG: impaired fasting glucose; IGT: impaired glucose tolerance; DM: diabetes mellitus)

	ALL (*n* = 435)	Cortisol cut-off 1 µg ACTH test ≤ 180 µg/L		*p*	Cortisol cutoff 1 µg ACTH test ≤ 127 µg/L		*P*
		HYPO-HPA (*n* = 229)	NORMO-HPA (*n* = 206)		HYPO-HPA (*n* = 53)	NORMO-HPA (*n* = 382)	
Male gender, n (%)	236 (54.3)	**143 (62.4)**	**93 (45.1)**	**0.0004**	**40 (75.5)**	**196 (51.3)**	**0.002**
Age (yrs)	58.5 [20.3]	**55.4 [20.3]**	**60.7 [19.5]**	**0.003**	58.7 [17.3]	58.5 [20.5]	0.81
BMI (kg/m^2^)	27.4 [6.7] (*n* = 183)	27.3 [6.6] (*n* = 117)	27.5 [7.0] (*n* = 66)	0.86	28.4 [6.6] (*n* = 23)	27.2 [6.8] ] (*n* = 160)	0.44
Morning cortisol (µg/L)	111.5 [49.0]	**93.3 [46.3]**	**124.2 [39.3]**	**< 0.0001**	**55.0 [53.5]**	**114.0 [43.2]**	**< 0.0001**
Cortisol at + 30 min from ACTH 1 µg (µg/L)	170.0 [58.5]	**144.0 [44.5]**	**201.4 [37.8]**	**< 0.0001**	**85.0 [50.0]**	**178.0 [49.0]**	**< 0.0001**
Cortisol at + 60 min from ACTH 1 µg (µg/L)	153.6 [60.0]	**128.0 [42.5]**	**186.0 [42.9]**	**< 0.0001**	**85.0 [46.2]**	**160.5 [53.1]**	**< 0.0001**
Cortisol peak (µg/L)	178.0 [55.1]	**150.8 [36.6]**	**207.0 [35.3]**	**< 0.0001**	**98.0 [59.1]**	**182.0 [49.1]**	**< 0.0001**
Glucose (mg/dL)	89.5 [21.0] (*n* = 262)	**87.0 [19.8]** **(n = 155)**	**94.0 [22.0]** **(n = 107)**	**0.0003**	**87.0 [14.5]** **(n = 36)**	**91.0 [22.0]** **(n = 226)**	**0.0006**
Sodium (mmol/L)	141.0 [4.0] (*n* = 329)	**141.0 [4.0]** **(n = 181)**	**142.0 [4.0]** **(n= 148)**	**0.0008**	**140.5 [3.5]** **(n= 44)**	**141.0 [2.0]** **(n = 285)**	**0.03**
Potassium (mmol/L)	4.2 [0.4] (*n* = 308)	4.2 [0.4] (*n* = 168)	4.3 [0.4] (*n* = 140)	0.58	4.2 [0.4] (*n* = 41)	4.2 [0.4] (*n* = 267)	0.83
Pituitary lesion, n (%)	396 (91.0)	210 (91.7)	186 (90.3)	0.62	50 (94.3)	346 (90.6)	0.61
Type of pituitary lesion, n (%)				0.15			0.07
Non-functioning pituitary adenoma	151 (38.1)	86 (41.0)	65 (35.0)		25 (50.0)	126 (36.4)	
Functioning pituitary adenoma	133 (33.6)	66 (31.4)	67 (36.0)		12 (24.0)	121 (35.0)	
Craniopharyngioma and Rathke’s cleft cyst	20 (5.1)	12 (5.7)	8 (4.3)		4 (8.0)	16 (4.6)	
Meningioma	40 (10.1)	15 (7.1)	25 (13.4)		1 (2.0)	39 (11.3)	
Other types of pituitary lesions	52 (13.1)	31 (14.8)	21 (11.3)		8 (16.0)	44 (12.7)	
Lesion diameter (cm)	2.2 [1.5] (*n* = 332)	2.1 [1.4] (*n* = 173)	2.2 [1.5] (*n* = 159)	0.78	**2.5 [1.2]** **(n = 45)**	**2.1 [1.5]** **(n = 287)**	**0.04**
Neurosurgery, n (%)	290 (66.7)	148 (64.6)	142 (68.9)	0.40	37 (69.8)	253 (66.2)	0.72
Radiotherapy, n (%)	122 (28.0)	59 (25.8)	63 (30.6)	0.31	9 (17.0)	113 (29.6)	0.08
Time between radiotherapy and ACTH test performance (yrs)	5.1 [6.0] (*n* = 119)	5.1 [6.0] (*n* = 57)	4.8 [6.2] (*n* = 62)	0.60	3.0 [2.4] (*n* = 9)	5.2 [6.2] (*n* = 110)	0.28
Other pituitary deficits, n (%)	223 (51.3)	127 (55.5)	96 (46.6)	0.08	**39 (73.6)**	**184 (48.2)**	**0.0009**
Panhypopituitarism, n (%)	36 (8.3)	**26 (11.4)**	**10 (4.9)**	**0.02**	**13 (24.5)**	**23 (6.0)**	**< 0.0001**
Number of other pituitary deficits, n (%)				**0.02**			**< 0.0001**
None	212 (48.7)	**102 (44.6)**	**110 (53.4)**		**14 (26.4)**	**198 (51.8)**	
One	118 (27.1)	**58 (25.3)**	**60 (29.1)**		**11 (20.7)**	**107 (28.0)**	
Two	69 (15.9)	**43 (18.8)**	**26 (12.6)**		**15 (28.3)**	**54 (14.1)**	
Three	33 (7.6)	**23 (10.0)**	**10 (4.9)**		**10 (18.9)**	**23 (6.1)**	
Four	3 (0.7)	**3 (1.3)**	**0 (0.0)**		**3 (5.7)**	**0 (0.0)**	
Type of other pituitary deficits, n (%)							
Central hypothyroidism	138 (31.7)	**84 (36.7)**	**54 (26.2)**	**0.03**	**32 (60.4)**	**106 (27.7)**	**< 0.0001**
Central hypogonadism	135 (31.0)	77 (33.6)	58 (28.2)	0.26	**25 (47.2)**	**110 (28.8)**	**0.01**
Growth hormone deficiency	74 (23.1) (*n* = 321)	**49 (28.5)** **(n = 172)**	**25 (16.8)** **(n = 149)**	**0.02**	**19 (45.2)** **(n = 42)**	**55 (19.7)** **(n = 279)**	**0.0005**
Central diabetes insipidus	20 (4.6)	15 (6.6)	5 (2.4)	0.07	**7 (13.2)**	**13 (3.4)**	**0.005**
Impairment of glucose metabolism (IFG, IGT or DM), n (%)	125 (28.7)	**56 (24.4)**	**69 (33.5)**	**< 0.05**	11 (20.8)	114 (29.8)	0.23

### Patient characteristics according to different peak cortisol responses to the ACTH test for the diagnosis of CAI

Based on a cortisol peak ≤ 180 µg/L after 1 µg ACTH stimulation, 229 patients were diagnosed with CAI (HYPO-HPA), while 206 were classified as having normal HPA function (NORMO-HPA). HYPO-HPA patients were younger (*p* = 0.01) and more frequently male (*p* < 0.001). Pituitary lesion characteristics and treatments were similar across groups. While overall hypopituitarism (defined by the presence of at least one other pituitary deficit besides CAI) prevalence, did not differ significantly, panhypopituitarism was more common in HYPO-HPA (*p* = 0.02). Moreover, in the HYPO-HPA group, other pituitary deficits besides CAI were more frequently present compared to the NORMO-HPA group, both in terms of the absolute number of associated deficits (*p* = 0.02) and when considering individual hypothalamic-pituitary axis deficiencies, at least for the thyroid (*p* = 0.03) and somatotropic axes (*p* = 0.02). Serum sodium (*p* < 0.001) and glucose (*p* < 0.001) were lower in HYPO-HPA, with fewer glucose metabolism disorders (defined as impaired fasting glucose, impaired glucose tolerance, or diabetes mellitus ) (*p* < 0.05) (Table 1).

Using a cortisol peak response ≤ 127 µg/L to the 1 µg ACTH test as the criterion for defining CAI, 53 patients were HYPO-HPA, while 382 patients were considered NORMO-HPA.

With this classification, all previously observed differences between the HYPO-HPA and NORMO-HPA groups were confirmed, except for age and glucose metabolism impairment, which no longer showed a significant difference between the two groups (Table 1). Furthermore, under this new classification, not only did panhypopituitarism remain more frequent in the HYPO-HPA group (*p* < 0.001), but the higher prevalence of any degree of hypopituitarism in HYPO-HPA compared to NORMO-HPA also reached statistical significance (*p* < 0.001). Additionally, all individual pituitary deficiencies were more frequent in the HYPO-HPA group than in the NORMO-HPA group. Although no statistically significant difference was observed in the overall prevalence of pituitary lesions, even with this classification, the prevalence of specific lesion types approached statistical significance (*p* = 0.07), driven by a trend toward a higher prevalence of non-functioning pituitary adenomas, craniopharyngiomas, and Rathke’s cleft cysts in the HYPO-HPA group compared with the NORMO-HPA group. Finally, and notably, with this classification, the size of the pituitary lesion potentially underlying the hormonal impairment was also found to be significantly greater in the HYPO-HPA group than in the NORMO-HPA group (*p* = 0.04), whereas this difference had not been observed in the previous classification (Table 1).

### Low-dose ACTH test results based on the application of different peak cortisol cut-offs for the diagnosis of CAI

In the overall cohort, basal cortisol was significantly correlated with cortisol levels at + 30 min (*r* = 0.53), + 60 min (*r* = 0.59), and peak (*r* = 0.56) during the low-dose ACTH test (all *p* < 0.001), as well as with age (*r* = 0.13, *p* = 0.01). Cortisol peak also correlated with age (*r* = 0.15, *p* = 0.01), and with cortisol at + 30 min (*r* = 0.92) and + 60 min (*r* = 0.86) (all *p* < 0.001).

Morning serum cortisol levels were significantly lower in HYPO-HPA than in NORMO-HPA (*p* < 0.001), regardless of the peak cortisol threshold used in the 1 µg ACTH test to define the presence of CAI (Table 1). The low-dose ACTH test significantly stimulated cortisol secretion in both HYPO-HPA (*p* < 0.001) and NORMO-HPA (*p* < 0.001) patients, independently of the cortisol peak threshold applied in the 1 µg ACTH test. The mean cortisol curve during the low-dose ACTH test was significantly higher in the NORMO-HPA group than in the HYPO-HPA group at every time point (*p* < 0.001), regardless of the threshold used to define CAI in the 1 µg ACTH.

### Multivariate logistic regression analysis of predictors of CAI

The results of the multivariate logistic analysis are summarized in Table 2.

**Table 2:** Multivariate analysis model of predictors associated with the development of hypothalamic-pituitary-adrenal (HPA) axis deficiency, as defined by a cortisol peak ≤ 180 µg/L (A), or ≤ 127 µg/L (B) during a low-dose ACTH stimulation test, in patients with a history of pituitary disease

HPA DEFICIT	OR	95% C.I.	*p* value
**A**			
Morning basal cortisol levels	0.97	0.96–0.98	< 0.0001
Serum sodium levels	0.86	0.78–0.94	0.0007
Number of observations = 329, LR chi^2^ (2) = 96.36, *p* < 0.0001, Log likelihood = 356.415			
**B**			
Morning basal cortisol levels	0.95	0.93–0.97	< 0.0001
Male gender	2.75	0.91–8.31	0.07
Age	1.03	0.99–1.07	0.07
Central hypothyroidism	2.76	0.99–7.70	0.05
Serum glucose levels	0.98	0.96–1.01	0.18
Serum sodium levels	0.75	0.61–0.93	0.008
Number of observations = 231, LR chi^2^ (6) = 82.183, p < 0.0001, Log likelihood = 107.291			

C.I.: confidence interval; LR: likelihood ratio; OR: odds ratio

Using a cortisol peak response ≤ 180 µg/L to the 1 µg ACTH test as the criterion for defining CAI, morning baseline cortisol levels (OR 0.97, *p* < 0.001) and serum sodium levels (OR 0.86, *p* < 0.001) emerged as negative predictors of CAI in the multivariate logistic analysis.

When applying a cortisol peak response ≤ 127 µg/L to the 1 µg ACTH test, morning baseline cortisol levels (OR 0.95, *p* < 0.001) and sodium levels (OR 0.75, *p* = 0.01) remained negative predictors of CAI. In addition, age (OR 1.03, *p* = 0.07), male sex (OR 2.75, *p* = 0.07), and the presence of central hypothyroidism (OR 2.76, *p* = 0.05) were positively associated with CAI, although the associations did not reach full statistical significance.

### ROC curve analysis identifying basal morning cortisol levels that confirm or exclude CAI according to different peak cortisol responses to the ACTH test

**Fig. 1:**
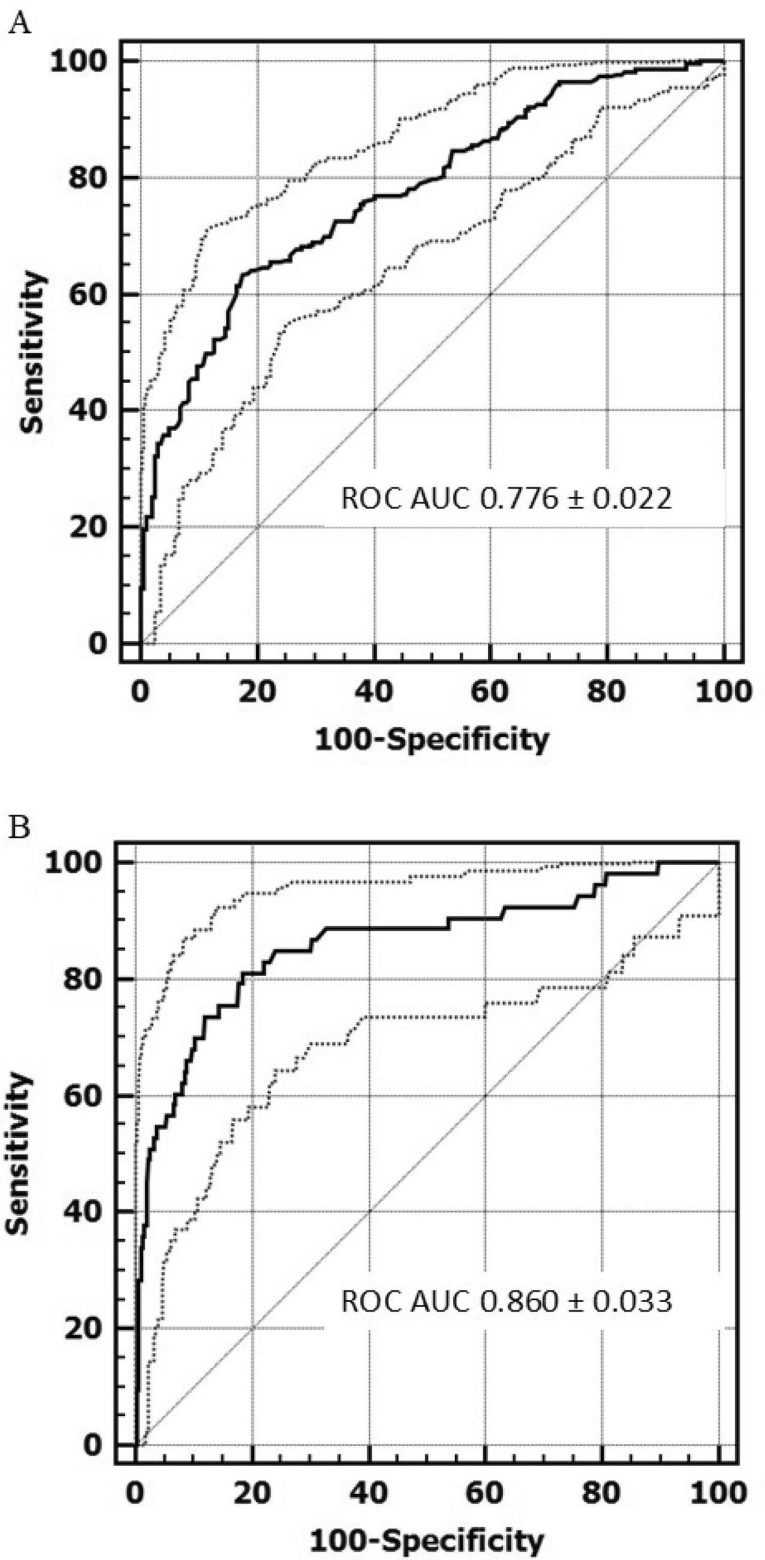
ROC curve analyses of morning serum cortisol levels for the diagnosis of hypothalamic-pituitary-adrenal (HPA) axis deficiency in patients with a history of hypothalamic–pituitary disease. Central adrenal insufficiency was defined as a cortisol peak ≤ 180 µg/L (**A**), and ≤ 127 µg/L (**B**) during a low-dose ACTH stimulation test (dotted lines indicate confidence intervals).

**Fig. 2:**
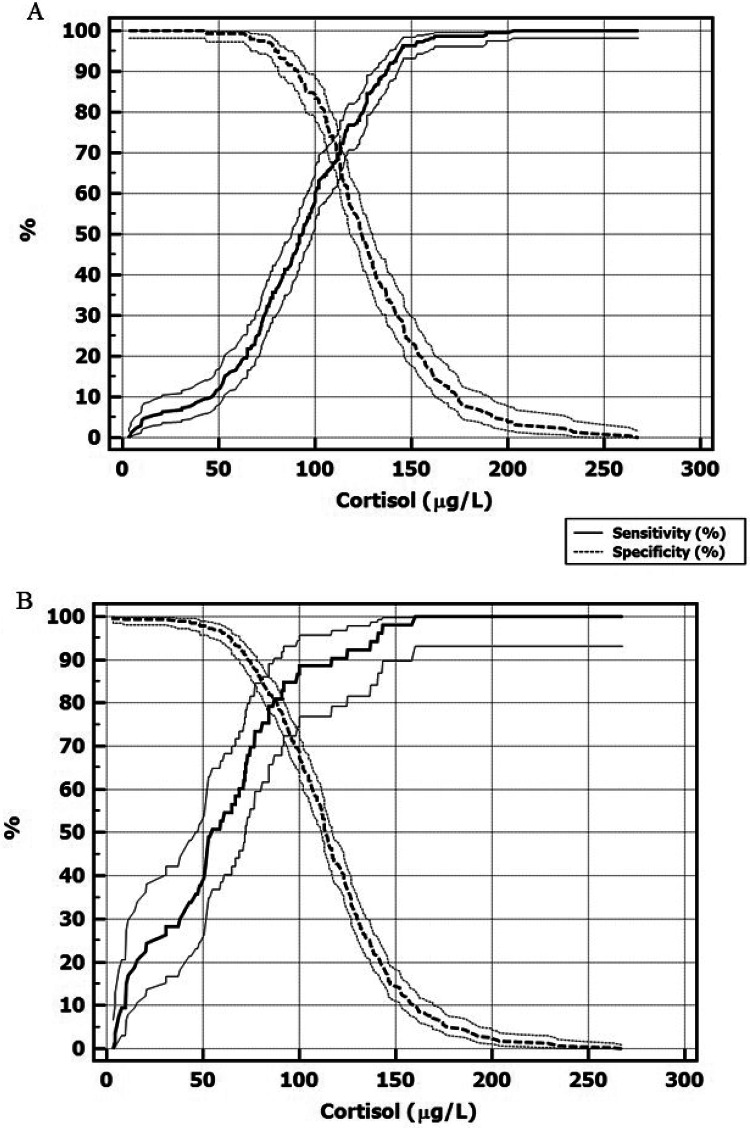
Sensitivity and specificity of morning serum cortisol at varying cut-off points, with central adrenal insufficiency defined as a cortisol peak ≤ 180 µg/L (**A**), and ≤ 127 µg/L (**B**) during a low-dose ACTH stimulation test (dotted lines indicate confidence intervals).

Using a cortisol peak ≤ 180 µg/L after 1 µg ACTH test as the CAI threshold, the optimal morning cortisol cut-off for discriminating between HPA deficiency and not, defined as the one with the best SE and SP, was ≤ 102 µg/L (SE 63.3%, SP 82.5%, LHR + 3.62, LHR- 0.44, ROC AUC 0.776 ± 0.022) (Figs. 1, 2; Table 3).

**Table 3:** Characteristics of the different morning basal cortisol cut-offs identified through ROC curve analysis in patients classified as having central adrenal insufficiency (CAI), based on their response to the 1-µg ACTH stimulation test using the two different cortisol peak thresholds reported in the literature

Cortisol Peak during 1 µg ACTH Test for the Diagnosis of CAI	Characteristics of sensitivity (SE) and specificity (SP) for the specific cut-off	Cut-off (µg/L)	SE (%)	SP (%)	PPV (%)	NPV (%)	LHR+	LHR-	ROC AUC
≤ 180 µg/L	Cut-off with SP ≥ 95%	≤ 80.8	37.1	95.2	89.5	57.7	7.65	0.66	0.776 ± 0.022
	Cut-off that combines the highest SE with the highest SP	≤ 102	63.3	82.5	80.1	66.9	3.62	0.44	
	Cut-off with SE ≥ 95%	≤ 144	95.6	29.6	60.2	85.9	1.36	0.15	
≤ 127 µg/L	Cut-off with SP ≥ 95%	≤ 60.9	54.7	96.3	67.4	93.9	14.9	0.47	0.860 ± 0.033
	Cut-off that combines the highest SE with the highest SP	≤ 87	81.1	81.4	37.7	96.9	4.37	0.23	
	Cut-off with SE ≥ 95%	≤ 141	96.2	21.2	14.5	97.6	1.22	0.18	

**Fig. 3:**
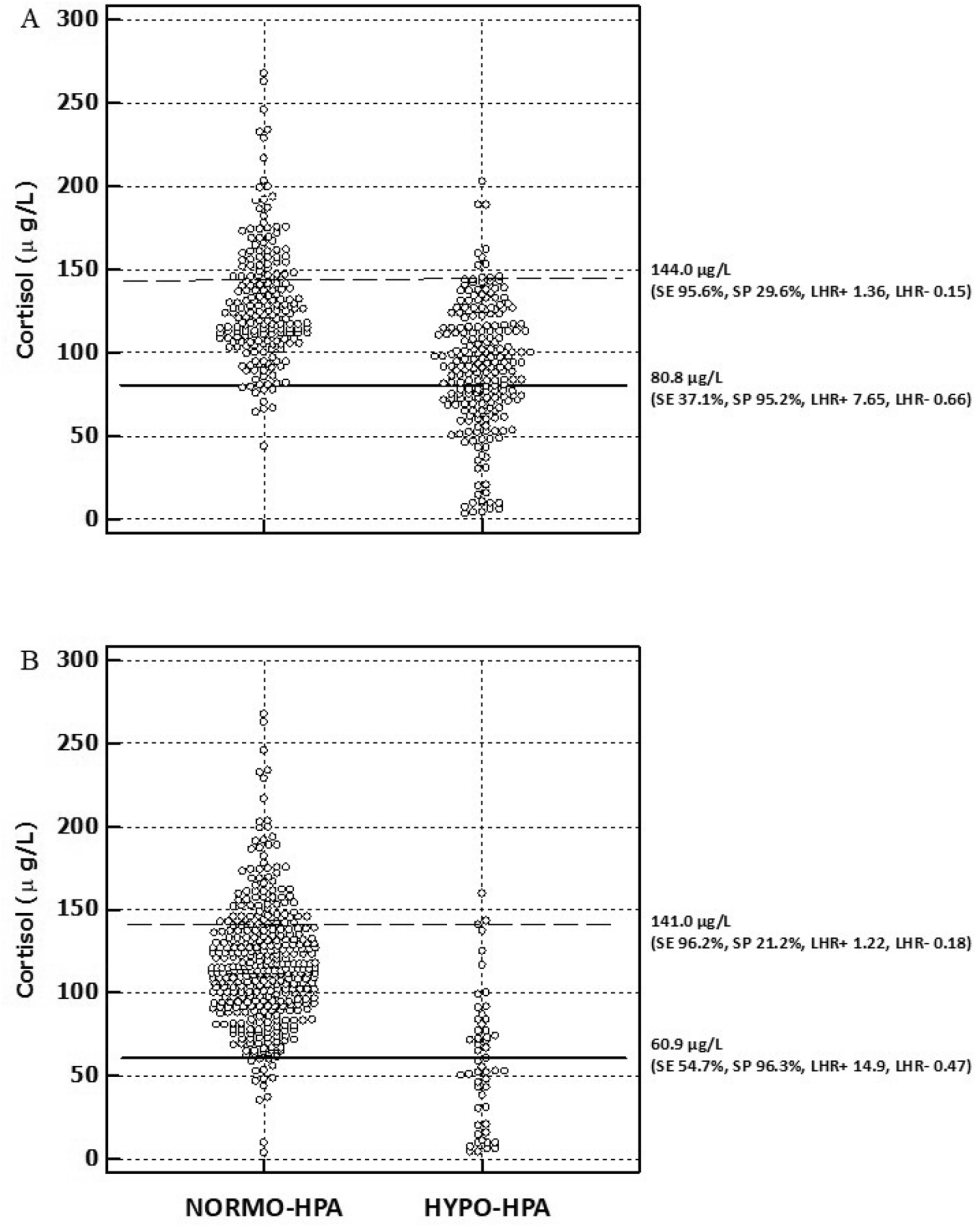
Individual morning serum cortisol values compared to the cortisol level that best predicts a deficient (continuous line) or normal (dashed line) response to a low-dose ACTH stimulation test in patients with (HYPO-HPA) and without (NORMO-HPA) central adrenal insufficiency, as defined by a cortisol peak ≤ 180 µg/L (**A**), and ≤ 127 µg/L (**B**) during a low-dose ACTH test (SE: sensitivity; SP: specificity; LHR+: positive likelihood ratio; LHR−: negative likelihood ratio).

For predicting a deficient response to the low-dose ACTH test, the best cut-off of morning serum cortisol was ≤ 80.8 µg/L (SE 37.1%, SP 95.2%, LHR + 7.65, LHR- 0.66) (Figs. 2, 3; Table 3). Applying this cut-off in our population would have avoided 95 tests, correctly classifying 85 patients but misclassifying 10. By contrast, the guideline cut-off (< 30 µg/L) would have avoided only 15 tests, with no misclassification.

The cut-off of morning serum cortisol concentration that best predicted a normal response to low-dose ACTH test was > 144 µg/L (SE 95.6%, SP 29.6%, LHR + 1.36, LHR- 0.15) (Figs. 2, 3; Table 3). Applying this cut-off in our population would have avoided 71 tests, correctly classifying 61. Compared to the guideline cut-off (> 150 µg/L), this would have saved 14 more tests but misclassified 3 patients; the guideline cut-off would have misclassified 8 with latent ACTH deficiency.

Using a cortisol peak response ≤ 127 µg/L to the 1 µg ACTH test as the criterion for defining CAI, the optimal morning cortisol cut-off for discriminating between HPA deficiency and not, was ≤ 87 µg/L (SE 81.1%, SP 81.4%, LHR + 4.37, LHR- 0.23, ROC AUC 0.860 ± 0.033) (Figs. 1, 2; Table 3), while a value ≤ 60.9 µg/L best predicted ACTH test failure (SE 54.7%, SP 96.3%, LHR + 14.9, LHR- 0.47) (Figs. 2, 3; Table 3). Applying this cut-off in our population would have avoided 43 tests, correctly classifying 29 patients with 14 false positives. Using the guideline cut-off (< 30 µg/L), only 15 low-dose ACTH tests would have been avoided, while 2 patients would have been incorrectly classified as pathological, despite not being affected by CAI.

The cut-off of morning serum cortisol concentration that best predicted a normal response to low-dose ACTH test was > 141 µg/L (SE 96.2%, SP 21.2%, LHR + 1.22, LHR- 0.18) (Figs. 2, 3 ; Table 3), reducing tests by 26 vs. the guideline, with only one misclassified case. Applying the guidelines’ cut-off > 150 µg/L would have led to the incorrect classification of only 1 subject with latent ACTH deficiency as HPA sufficient.

Table 3 summarizes diagnostic performance across cut-offs and thresholds.

## Discussion

To our knowledge, this is the first study to propose novel morning cortisol cut-offs for confirming or excluding CAI, measured with the Roche Elecsys^®^ Cortisol II assay — a next-generation monoclonal antibody immunoassay with improved analytical specificity compared with older assays used to establish previous thresholds. Our findings support the use of morning serum cortisol as a reliable, simple, and cost-effective tool for diagnosing CAI. Specifically:

- The low-dose ACTH test robustly stimulates the HPA axis in both NORMO-HPA and HYPO-HPA patients, regardless of the peak cortisol level used to define CAI.
- Morning cortisol correlates positively with HPA axis response to ACTH, confirming its diagnostic value, as shown by ROC AUC analysis.
- Between literature-proposed peak cortisol thresholds (≤ 180 or ≤ 127 µg/L), the 127 µg/L cut-off best clinically distinguishes CAI from preserved HPA axis function.
- Using a cortisol peak ≤ 180 µg/L as the diagnostic threshold for CAI, a morning cortisol ≤ 80.8 µg/L predicts CAI with ≥ 95% specificity, whereas values > 144 µg/L exclude it with ≥ 95% sensitivity.
- Using a more specific threshold (cortisol peak ≤ 127 µg/L), a morning cortisol ≤ 60.9 µg/L identifies CAI with ≥ 95% specificity, whereas values > 141 µg/L exclude it with ≥ 95% sensitivity.
- Thus, a morning cortisol ≤ 60.9 and > 141 µg/L appear to be the most accurate for confirming or ruling out CAI when measured with the Roche Elecsys^®^ Cortisol II assay.

An accurate evaluation of HPA axis is essential for the management of patients with clinical suspect of CAI [12]. A proper and fast diagnosis of CAI is mandatory because undiagnosed ACTH deficiency is a life-threatening condition. Conversely, an inappropriate glucocorticoid replacement as chronic therapy, in the long run, has also demonstrated detrimental effects, such as increased body mass index, an increased incidence of type 2 diabetes mellitus and arterial hypertension, reduced bone mineral density and an increased risk of vertebral fractures [10, 11]. Therefore, considering the dramatic importance but also the risks of glucocorticoid replacement therapy, the correct diagnosis of CAI is pivotal [6].

The measurement of morning serum cortisol is the initial step to diagnose CAI; however, when serum cortisol levels are not clearly sufficient or insufficient to respectively exclude or confirm adrenal insufficiency, dynamic testing is recommended.

However, it remains unclear if a defined morning serum cortisol level could reliably predict an adequate response to stimulation test, thus avoiding a more complex diagnostic pathway.

The Endocrine Society clinical practice guideline for the diagnosis and treatment of hypopituitarism suggests that a morning serum cortisol level < 30 µg/L is indicative of CAI and a morning serum cortisol level > 150 µg/L likely excludes the diagnosis, but underlines that the quality of evidence is very low [12]. Therefore, the serum cortisol cut-off that excludes the need of further investigation is uncertain. In the literature, several studies aimed to identify morning baseline cortisol levels that can predict the response to the ACTH test, but most are focused on the standard-dose test (250 µg) [14–20], while studies specifically oriented towards identifying such cut-off values in relation to the low-dose ACTH test (1 µg) [21, 22] are considerably more limited. In this regard, Perton and Colleagues [21] identified an upper morning cortisol cut-off of 135.9 µg/L and a lower cut-off of 52.6 µg/L. These cut-offs demonstrated the highest sensitivity and negative predictive value and the highest specificity and positive predictive value, respectively, with the ultimate aim of safely reducing the number of patients requiring the low-dose ACTH test. Other Authors [22] proposed a morning serum cortisol cut-off < 33 µg/L as the best predictor of a deficient response to the ACTH test, while a value > 138 µg/L had the highest sensitivity for excluding adrenal insufficiency. However, this latter study included not only patients who underwent the low-dose ACTH test but also those who underwent the standard-dose test, who constituted approximately 55% of the cohort [22]. Moreover, it should be noted that both of these studies used a cortisol response cut-off of 180 µg/L for the ACTH test, a threshold derived from earlier studies employing older assay methods, whereas newer, specific monoclonal antibody immunoassays may have lower diagnostic thresholds.

In this context, our study aimed to identify the most accurate morning basal cortisol values for confirming or excluding the diagnosis of CAI, using the 1 µg ACTH test as the gold standard. We compared the results obtained when varying the cut-off used to interpret the ACTH test, moving from the traditional threshold of 180 µg/L to the more recent cut-offs of 127 µg/L. This newer threshold has been proposed by recent studies employing second-generation cortisol assay methods, which are more sensitive and specific, and correspond to the method currently used in our laboratory.

Our study supports a narrow range of morning serum cortisol cut-off able to predict the diagnosis of CAI and increases the quality of evidence on this topic. In particular, in the present study, conducted on a cohort of 435 patients, we generated ROC curves to evaluate the diagnostic performance of various basal cortisol values, using different diagnostic criteria based on peak cortisol cut-offs (≤ 180 µg/L, and ≤ 127 µg/L) in response to the 1 µg ACTH test. Based on the ROC AUCs, SE, SP, and LHRs of each morning cortisol cut-off pair derived from the two ACTH test criteria, the most accurate thresholds were those defined using a cortisol peak ≤ 127 µg/L as the diagnostic criterion. In this context, the superior performance of this diagnostic threshold is further supported by the analysis of the characteristics of the HYPO-HPA and NORMO-HPA groups defined according to this criterion. These two populations differ not only in terms of morning basal cortisol levels and response to the ACTH test, but also across various clinical and laboratory parameters, suggesting a greater degree of impairment in the hypothalamic-pituitary-peripheral axis in the HYPO-HPA group.

When using a cortisol peak ≤ 127 µg/L as the reference criterion for CAI, a morning serum cortisol concentration ≤ 60.9 µg/L emerged as the best threshold for predicting CAI. This cut-off demonstrated a SP of 96.3%, PPV of 67.4%, and a LHR + of 14.9, with a corresponding ROC AUC of 0.860 ± 0.033. Application of this threshold would have avoided the need for stimulation testing in 43 patients, with 29 of them correctly classified as HPA-axis deficient. Although 14 false positives would have occurred, this trade-off—corresponding to 3.2% of the total study population—is acceptable in scenarios where high specificity is prioritized, especially to limit unnecessary dynamic testing.

Conversely, to exclude CAI, the morning cortisol cut-off > 141 µg/L, derived using the same cortisol peak criterion (≤ 127 µg/L), demonstrated a SE of 96.2%, NPV of 97.6%, and a LHR– of 0.18. This value would have eliminated 83 ACTH tests, correctly identifying 81 patients as HPA-axis sufficient. This threshold also outperformed the guideline-recommended value of > 150 µg/L in terms of test reduction and diagnostic accuracy, misclassifying only one patient with latent ACTH deficiency. Their use could lead to a more efficient diagnostic algorithm, substantially reducing the need for ACTH stimulation testing without compromising diagnostic accuracy.

It must be emphasized that the cut-offs identified are valid for the Roche Elecsys^®^ Cortisol II assay used in this study only; however, our study also allows a comparison (Bland-Altman plot) with other assays to determine potential correction factors. Lack of standardization across different platforms for cortisol measurement is an issue in routine clinical practice, because some cut-off values established with immunoassay could not be used with other platforms [6]. Liquid chromatography coupled with tandem mass spectrometry (LC-MS/MS) offers improved specificity and sensitivity; however, cortisol cut-off proposed in guidelines did not consider cortisol assays [6, 12, 32, 33].

Our study confirms previous findings [34–37] that the low-dose ACTH test effectively stimulates cortisol secretion in both individuals with normal and impaired HPA axis function, regardless of the peak cortisol threshold used to define CAI. We also observed significantly lower morning cortisol levels in CAI patients compared to those with normal HPA function, with a positive correlation to the cortisol peak during the low-dose ACTH test. These observations support the use of morning serum cortisol as a key screening tool for HPA axis function in hypothalamic-pituitary disease. Specifically, identifying a reliable morning cortisol threshold to predict ACTH test response provides a simple, safe, and cost-effective strategy to reduce unnecessary dynamic testing.

**Fig. 4:**
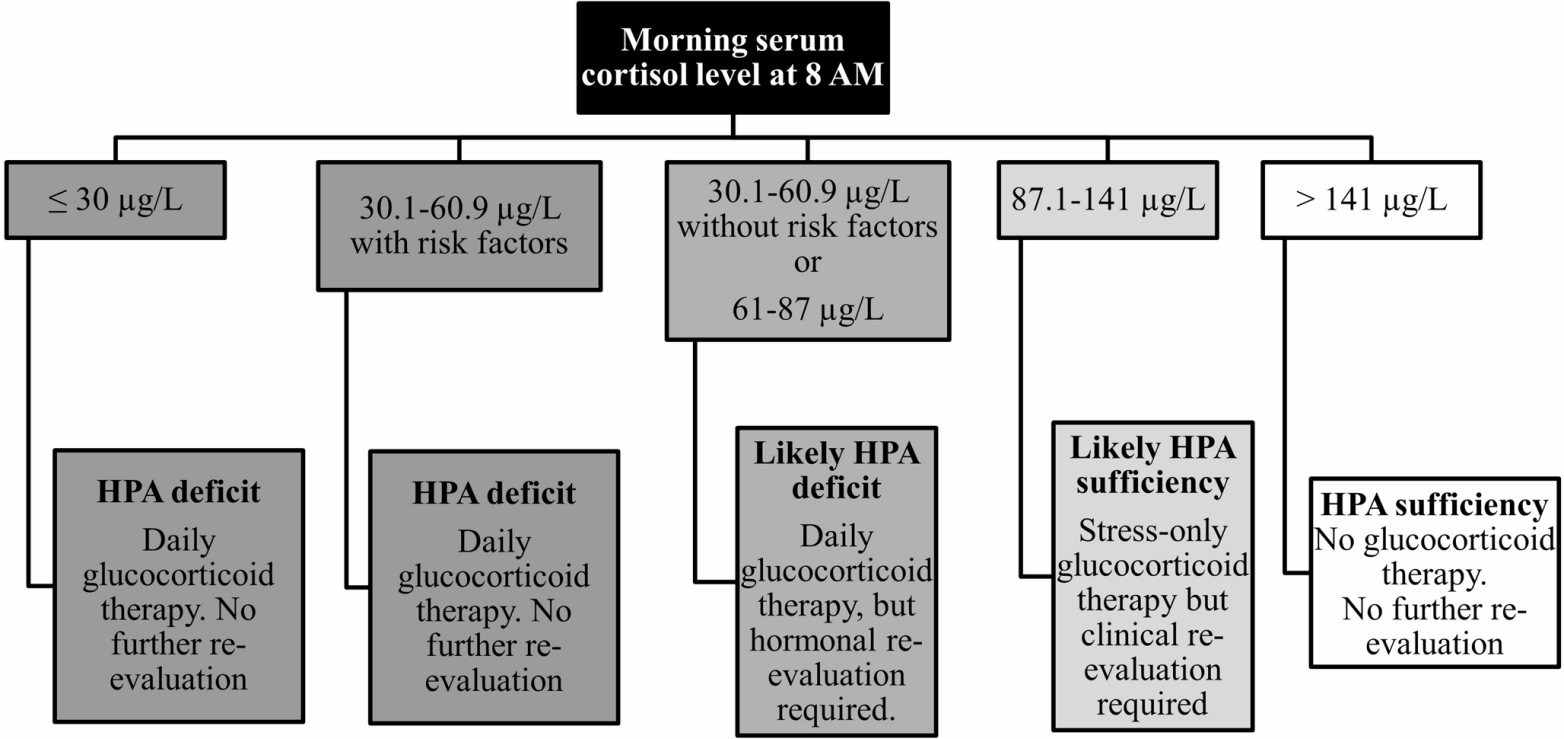
Algorithm for evaluating the hypothalamic-pituitary-adrenal (HPA) axis based on morning serum cortisol levels, as determined by ROC curve analysis. (Risk factors: at least one among: male sex, ≥ 1 pituitary deficiency, or hyponatriemia).

Based on these findings, we propose integrating morning serum cortisol into the diagnostic algorithm for CAI, as shown in Fig. 4. In our cohort, applying the proposed algorithm would have spared ACTH testing in 28% of patients (121/435) based on (i) morning cortisol ≤ 30 µg/L (15 patients), (ii) morning cortisol between 30.1 and 60.9 µg/L and at least one among male sex, ≥ 1 other pituitary deficit, or hyponatriemia (23 patients), (iii) morning cortisol > 141 µg/L (83 patients), indicating likely HPA deficiency or sufficiency, respectively. Specifically, 73.7% (28/38) of those with cortisol ≤ 30 µg/L or between 30.1 and 60.9 µg/L and at least one among the previous selected characteristics, and 97.6% (81/83) with > 141 µg/L were correctly classified by basal cortisol. An additional 54.7% of patients, with cortisol levels between 87.1 and 141 µg/L, would have been considered likely HPA sufficient, managed with glucocorticoid therapy only during stress and subject to clinical follow-up. Meanwhile, 17.5% of patients, with cortisol levels between 30.1 and 60.9 µg/L and without risk factors or between 61 and 87 µg/L, would have been considered likely HPA deficient and treated with chronic glucocorticoid therapy pending hormonal reassessment. Overall, this approach would reduce ACTH testing by 28% (121/435) versus 16.6% (72/435) using current guideline cut-offs alone.

The proposed diagnostic algorithm may contribute to improve cost-effectiveness. By allowing the safe exclusion or strong suspicion of CAI based on morning cortisol levels and predefined clinical-biochemical risk factors, the number of ACTH stimulation tests required is substantially reduced. This targeted use of dynamic testing lowers laboratory workload, minimizes patient inconvenience, and optimizes the use of clinical resources. Although a formal cost-effectiveness analysis was not within the scope of the present study, our findings suggest that a stratified approach could streamline the diagnostic process and potentially reduce the economic and logistical burden associated with the routine use of stimulation tests. However, it should be emphasized once again that the diagnostic flow-chart proposed in Fig. 4 is applicable only when using the Roche Elecsys^®^ Cortisol II assay.

Finally, we found, like in previous studies [38–40] and regardless of the peak cortisol cut-off used to define CAI, a higher prevalence of men in the group of subjects with CAI; this observation cannot be attributed to a higher prevalence of neurosurgery and/or radiotherapy in males, as the difference observed compared to females was not statistically significant (data not shown); similarly, the prevalence of different type of pituitary lesions and their maximum dimensions were not statistically different between males and females (data not shown). All these observations suggest that male sex may represent a possible independent risk factor for CAI, although this hypothesis requires confirmation in specifically designed studies.

In this regard, it is worth highlighting that, in multivariate analysis—and regardless of the cortisol peak threshold used to define CAI during the 1 µg ACTH stimulation test—morning basal cortisol levels and serum sodium consistently emerged as negative predictors of CAI. Additionally, central hypothyroidism and male sex approached significance using the 127 µg/L threshold. These findings support the development of a clinical-laboratory score incorporating cortisol, sodium, sex, and pituitary function to better predict CAI—offering a safer and simpler alternative to dynamic testing [40].

Our study presents some strengths and limitations. The major strengths are the rigorous inclusion of only patients with a history of hypothalamic-pituitary disease to limit the investigation to the appropriate clinical context, the high number of studied subjects, and the exclusion of conditions that could potentially confound the test results.

The main limitations of this study include its retrospective design. In addition, the proposed basal cortisol cut-offs are applicable only when the same pre-analytical conditions and analytical method are used. Consequently, the suggested thresholds should not be directly extrapolated to different sampling procedures or assay platforms. Moreover, further prospective validation in independent cohorts will be essential before these thresholds can be reliably translated into clinical practice.

In conclusion, our data define new morning cortisol cut-offs (≤ 60.9 µg/L and > 141 µg/L) using the Roche Elecsys^®^ Cortisol II assay, which can reliably confirm or exclude CAI and minimize the need for dynamic testing. These thresholds, derived using second-generation assays, significantly reduce the diagnostic “grey zone” and enhance clinical decision-making. The improved specificity of modern assays supports the adoption of updated, clinically meaningful reference values. While prospective validation is needed, our findings show that robust diagnostic thresholds can be established independently of assay-related numerical differences.

## Data Availability

The data sets generated and analyzed during the current study are not publicly available but are available from the corresponding author on reasonable request.
